# Smart Sensors Applications for a New Paradigm of a Production Line

**DOI:** 10.3390/s19030650

**Published:** 2019-02-05

**Authors:** Marina Indri, Luca Lachello, Ivan Lazzero, Fiorella Sibona, Stefano Trapani

**Affiliations:** 1 Dipartimento di Elettronica e Telecomunicazioni, Politecnico di Torino, Corso Duca degli Abruzzi 24, 10129 Torino, Italy; ivan.lazzero@polito.it (I.L.); fiorella.sibona@polito.it (F.S.); 2 COMAU SpA, Via Rivalta 30, 10095 Grugliasco, Italy; luca.lachello@comau.com (L.L.); stefano.trapani@comau.com (S.T.); 3 Dipartimento di Automatica e Informatica, Politecnico di Torino, Corso Duca degli Abruzzi 24, 10129 Torino, Italy

**Keywords:** Industry 4.0, production line, smart sensor applications

## Abstract

Industrial plants are going to face a deep renewing process within the Industry 4.0 scenario. New paradigms of production lines are foreseen in the very near future, characterized by a strict collaboration between humans and robots and by a high degree of flexibility. Such envisaged improvements will require the smart use of proper sensors at very different levels. This paper investigates three different aspects of this industrial renewing process, based on three different ways of exploiting sensors, toward a new paradigm of a production line. The provided contributions, offering various types of innovation and integration, are relative to: (i) a virtual sensor approach for manual guidance, increasing the potentialities of a standard industrial manipulator, (ii) a smart manufacturing solution to assist the operator’s activity in manual assembly stations, through an original exploitation of multiple sensors, and (iii) the development of an advanced robotic architecture for a flexible production line, in which a team of autonomous mobile robots acts as a meta-sensor net supporting traditional automated guided vehicles. Accurate analyses of existing state-of-the-art solutions compared with the proposed ones are offered for the considered issues.

## 1. Introduction

Industry in the 21st Century is undergoing a deep transformation in its inner conception. The possibility of managing big amounts of data at low cost, together with the almost infinite data processing capacity, is leading to industrial plants free from classical, rigid constraints, which are no longer considered as insurmountable. Furthermore, the need to transfer huge quantities of information in a fast and reliable way demands advanced communication methods, drawing extensive research interest in technologies that meet the increasingly stringent requirements of specific industrial applications. Indeed, the leading wireless networks, based on new technologies such as 5G, represent the upcoming solution to the issues of performance requirements [1]. In this new scenario, the big challenge is becoming the production of custom versions of a certain product, in quantities that may change on demand, relying on an enhanced flexibility of the production lines [2,3]. Various examples of ideal systems projected over the next decade can be found, e.g., the multi-silhouette production line by PSA [4], as well as pilot plants, like the one proposed by Kuka in [5], based on the matrix production paradigm, which represents a recent effort to break down the conventional linked production line into standardized and categorized manufacturing cells, placed along a grid layout.

The main components of the production line are uprooted from their fixed position and role: industrial manipulators enter the human operator working space in the guise of collaborative robots (or cobots); the traditional Automated Guided Vehicle (AGV) becomes smart, autonomously finds its way through the distributed working stations, and becomes increasingly intelligent, turning into the present-day Autonomous Mobile Robot (AMR), which is gradually taking a fundamental role in the new dynamic and productivity-oriented industrial environment, in which flexibility leads the production line development. Service robotics brought in innovative technologies and solutions that are slowly migrating to the industrial sector, where smart robots will be able to learn tasks without formal programming and to cooperate autonomously with other smart devices and factory workers [6]. Mobile manipulators (i.e., robotic arms on mobile bases) are slowly entering warehouses and factories, making obsolete the idea of an industrial robot strictly associated with a fixed and caged manipulator: AMRs, cobots, and enhanced manual stations, fully integrated within the automated lines, are going to characterize the smart factories of the very near future (see, e.g., [7]).

**Figure 1:**
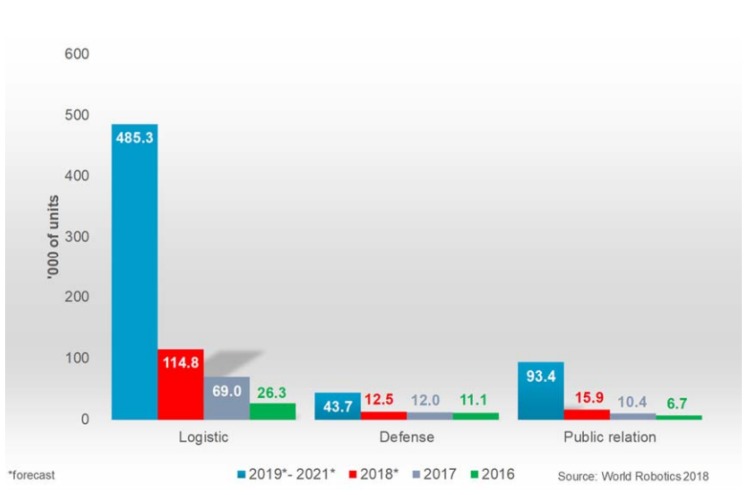
Service robots for professional use in main applications. Unit sales for 2016 and 2017 and forecast for 2018 and 2019–2021.

Although the concept of autonomous mobile robots is not new (the first generic AMR patent was from 1987 [8]), its application to an industrial context has come up in recent developments and is expected to increase significantly in the near future. In fact, logistic systems, e.g., AGVs, make up 66% of the total forecast of service robots (with different degrees of autonomy) from 2019–2021 (Figure 1) [9].

**Figure 2:**
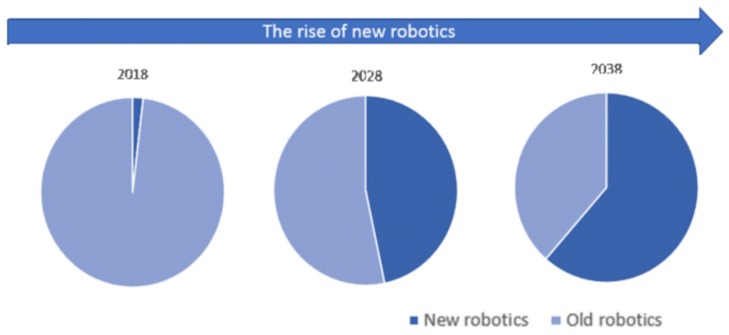
Long-term forecasts of the market evolution for new robotics according to the New Robotics and Drones IDTechEx report.

In particular, AMRs fall within the so-called new robots, i.e., robotic systems compliant with collaborative operations, characterized by a higher degree of autonomy and capable of autonomous decision making. According to a new IDTechEx research work [10] about new robotics and drones forecast for the 2018–2038 period, we will see a dramatic increase in new robotics deployment, with the support of decreasing hardware and software development costs (Figure 2).

All the envisaged improvements, leading to the smart factories of the Industry 4.0 scenario, have sensors and their proper usage as fundamental pillars [11]. The desired more adaptive production lines and the whole product life cycle have to be supported by a smart choice of heterogeneous sensors and/or a heterogeneous choice of smart sensors, through which not only the final goals can be achieved, but also a smooth transition can be set up from the current industrial standards toward the full Industry 4.0 reality.

In this paper, thanks to a strict academia-industry collaboration between Politecnico di Torino and COMAU S.p.A., the authors investigate three different aspects of this industrial renewing process, based on three different ways of exploiting sensors, aiming for a new paradigm of a production line, including enhancements that can be applied already today or tomorrow at the latest, allowing a smooth, successful transition toward the factories of the future.

The first contribution is relative to the increase of the potentialities of a traditional industrial manipulator through a sensor-less technology, based on the creation of a virtual sensor using physical devices already present. In particular, a non-collaborative robot, lacking of any force/torque sensor, is made able to be directly moved by the human operator in manual guidance sessions. This way, the programming and teaching process of the robot becomes easier and more user-friendly.

The second contribution is the SMARTMAN 4.0 solution, given by the application of multiple physical sensors and pointing systems to manual assembly stations, allowing a monitoring both with respect to the operators’ privacy and useful for ensuring compliance with the assembly best-practices, which gathers information for the continuous improvement of the ergonomics and the efficiency of the manual stations. With the proposed solution, even the manual work stations become completely aligned and synergistic, from a holistic point of view, with the automated lines and their digitization.

While the first two contributions are relative to solutions already fully implemented in practice, the third one is constituted by the architecture foreseen in an on-going project (HuManS—Human-centered Manufacturing Systems), relative to the whole concept of the new production line, in which AMRs act as mobile sensors, distributed and reallocated as a supporting net to traditional AGVs, so as to reduce as much as possible the need for infrastructural interventions. This way, the AMRs take on the role of meta-sensors, within an extremely flexible production line, including also AGVs and robots sharing spaces with human operators.

The descriptions of the three contributions are provided in Section 2, Section 3 and Section 4, respectively, together with the analysis of other state-of-the-art solutions available in the literature or in the industrial world. All the contributions, offering different types of innovation and integration, either vertical (i.e., requiring a detailed knowledge of the specific matter) or coming as the result of an evolved integration of several parts, share the common characteristic that the result is superior to the mere sum of the components, and they synergically converge to the goals of efficiency, continuous improvement, and minimization of infrastructural interventions that represent the most significant aspects of the Industry 4.0 scenario. Finally, Section 5 addresses some conclusive considerations about the described solutions and their possible applications.

## 2. Virtual Sensors for Robot Manual Guidance and Collision Detection

Classical industrial manipulators, not specifically built to collaborate with humans, are usually equipped with standard proprioceptive sensors only, i.e., sensors that measure physical quantities relative to the internal state of the robot, like encoders for the joint position measurement and motor current sensors. More advanced applications, which would require further external sensors (i.e., sensors measuring quantities relative to the environment or to the interaction of the robot with it, like force/torque sensors), can be successfully implemented using a virtual sensor approach, in which the information coming from the available sensors is exploited to enhance the knowledge of the robot behavior. In this kind of approach, a software layer, i.e., the so-called virtual sensor, provides new “measures”, which are computed using both the information acquired by the real sensors (e.g., joint positions and motor currents) and the knowledge of some physical characteristics of the robot [12]. Different approaches can be found in the literature to implement virtual sensors providing contact forces and moments acting on the robot, like those based on an observer of the external forces [13,14] or using a filtered dynamic model like in [15].

In order to give to the human operator the possibility to guide the motion of a non-collaborative manipulator directly during the programming phase, a manual guidance approach is proposed in this section, after an overall analysis of the available state-of-the-art solutions for collision detection and manual guidance. The effectiveness of the proposed approach is confirmed by the experimental results reported in the last part of the section.

### 2.1. State-of-the-Art

Collision detection and post-collision management are different issues, which can be properly combined to achieve interesting robotic applications, e.g., manual guidance, which is a good way to define user-friendly robot programming approaches (like programming by demonstration [16]) and collision reaction strategies that can avoid/reduce possible mechanical damage to the robot.

Collision Detection (CD) is a commonly-used term in the robotics field, to define those methodologies that are intended to detect collisions between the robot and the surrounding environment. CD algorithms are usually based on the idea of applying a threshold to a signal (the collision signal) that varies according to the external forces applied on the robot. The threshold can be constant or time varying, but in any case, the main issue is to obtain a proper collision signal. Specific sensors (that are usually not included in standard industrial robots), like force sensors, can be used to obtain a measure of the force applied on a specific point of the robot structure or torque sensors to measure the torques applied on the robot joints. Accurate physical models of the robot are usually exploited to clean the signal from the force/torque components due to the dynamics of the robot, so obtaining a collision signal actually corresponding to the applied external forces/torques. The sensors already included in the robot, like the motor encoders and the current sensors, can be alternatively used to obtain an estimate of the torques applied on the robot joints, or in general a signal that varies according to the external forces applied on the robot. The collision detection and manual guidance approaches based on this kind of solution have the advantage that no extra sensors are required for their implementation. Various techniques can be found in the literature. Initially, CD methods were designed to detect collisions in production systems, where robots could accidentally hit other objects/robots, due to programming errors or to the presence of unforeseen objects. In [17], a collision detection scheme based on a disturbance observer system has been presented, in which collisions are detected by setting up a set of thresholds for every joint. In [18], the authors presented a collision detection scheme with adaptive characteristics, based on a Finite State Machine (FSM) (whose states are a priori labeled as safe or unsafe), processing both real and dynamically-modeled joint currents. Once an unsafe state is detected, a time-varying threshold is applied to detect a collision. Statistical time series methods have also been developed to achieve detection and identification of faults in an aircraft skeleton structure [19]. The main advantage of those methods lies in their ability to use data to build mathematical models that represent the true dynamical system. Even if applied to a different context, such methodologies were exploited later to develop robot collision detection procedures based on fuzzy identification [20].

Some studies [21] introduced the notion of human pain tolerance to set an acceptable pain level for a human; such a level can be used as a threshold for CD algorithms to reduce the impact force, so allowing their adoption in a Human-Robot Collaboration (HRC) context. Different methodologies have then been developed with the purpose of being suitable for HRC applications. In [21], the difference between the dynamic model torques and the actual motor torques was used to obtain a reliable detection scheme. A more general approach was presented in [22], where the robot generalized momentum is exploited to define two functions: σ(t) and r(t), σ(t) being the collision detection signal, whose value raises when a collision occurs and rapidly returns to zero when the contact is lost. Information about the force direction or the link on which the collision took place is provided by r(t), called the collision identification signal. A closed control architecture was proposed in [23], using only motor currents and joint positions in order to define suitable thresholds for the detection scheme, while in [24], the authors tried to refine such an approach, by preventing or greatly reducing the probability of false alarms using an appropriate band-pass filter with a changing frequency window, so as to facilitate the distinction between collisions and false alarms. Computationally efficient methods based on fuzzy identification and time series modeling [20] can also be found, whose adoption does not require the explicit knowledge of the robot dynamic model. A training phase is however necessary, but offline training procedures are available.

Several works can be found on robot Manual Guidance (MG) methodologies, as well. In [25], the authors proposed an approach based on the adoption of force/torque sensors to implement a control scheme that imposes a specific velocity profile according to the sensor measures. Many classical control schemes can also be found, like force control [26], impedance control [27], and admittance control [28,29], as well as more advanced methodologies like adaptive admittance control schemes [30] and variable impedance control schemes [31]. A further interesting approach based on the adoption of vision systems was presented in [32]; in this case, the robot motion is obtained using the images provided by a camera. Sensor-less methodologies (i.e., that do not use force/torque sensors) can also be mentioned, like the ones based on the adoption of an observer of external forces [22] to achieve manual guidance.

### 2.2. The Proposed Sensor-Less Approach to Manual Guidance

A sensor-less methodology implementing virtual sensors to manage both collision detection and manual guidance sessions is proposed. Such an approach is defined as sensor-less since no external sensors are required; only information provided by the proprioceptive sensors, typically included in industrial robots, is used, i.e., position of the joints and current absorbed by the motors. The methodology includes: (i) a monitoring phase, which detects if a collision occurred and distinguishes if it was due to an accidental impact with the environment, during a non-collaborative application, or determined by an intended human-robot contact, and (ii) a post-impact phase, which imposes an appropriate reaction strategy: an MG algorithm when an intended human-robot contact is detected or a CD reaction when an accidental collision occurs. The proposed procedure requires heuristic approaches to choose suitable values for some of the involved parameters, which must be customized for the specific robot to which it is applied. The choices made in the experimental application of the procedure to a COMAU Racer 7-1.4, used as the test-bed, are provided throughout the general description of the procedure, each time immediately after the equations or policies employed by the procedure using those parameters. Such choices, even if experimentally developed in a particular case, can provide useful guidelines and suggestions for the application to other manipulators.

**Figure 3:**
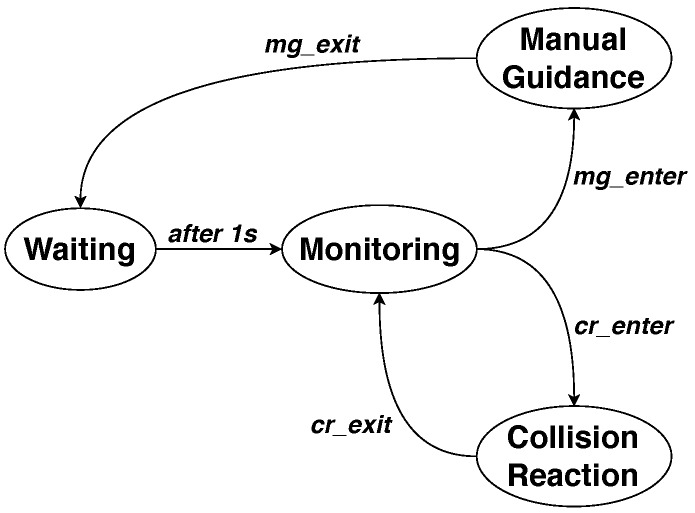
Basic state diagram for the state machine.

The finite state machine shown in Figure 3 manages all the phases of the developed procedure; it is composed of the following four states:

1. **Monitoring**: where the currents are monitored and decision parameters are updated. No orders are given to the robot for the position adjustment in this state.
2. **Manual Guidance**: where a manual guidance contact is detected and movement corrections are accordingly sent to the robot.
3. **Collision Reaction**: where a collision is detected and a reaction strategy is adopted to stop or move the robot back to a safe position.
4. **Waiting**: void state imposing the waiting of 1 s.

The algorithm starts from the **Monitoring** state, which refers to the normal operation of the robot during which no interactions occur. When an MG interaction is detected (i.e., condition *mg_enter* is satisfied), the system moves to the **Manual Guidance** state, and the MG interaction is enabled until condition *mg_exit* is met. At this point, the system moves to the **Waiting** state, where a 1-s delay is imposed before returning to the **Monitoring** state. The same operation occurs in the case of collision detection. Whenever condition *cr_enter* is met, the system moves to the **Collision Reaction** state and performs the programmed reaction strategy, before returning to the **Monitoring** state when condition *cr_exit* is satisfied.

#### 2.2.1. Monitoring State

The goal of the **Monitoring** state is to detect whether a collision occurred and to distinguish if it was due to the interaction between the user and the robot or an accidental collision, i.e., to verify if one of the conditions *mg_enter* and *cr_enter* is satisfied. In a sensor-less context, such a goal can be reached exploiting the information included in the residual current vector $I_{res}$ defined as:

(1) $$I_{res}\left(t\right)=I_{m}\left(t\right)-K_{t}^{-1}\tau_{dm}\left(t\right)$$

where $I_{m}\left(t\right)$ is the vector containing the currents measured on each motor, $K_{t}$ is the diagonal matrix of the motor torque constants, and $\tau_{dm}\left(t\right)$ is the motor torques vector computed using the available robot dynamic model. Using the matrix $K_{t}$, it is possible to obtain the vector of residual torques as:

(2) $$\tau_{res}\left(t\right)=K_{t}I_{res}\left(t\right)$$

In the absence of external forces applied to the robot, (2) highlights the error between the real measured torque and the value provided by the adopted dynamic model. Even if such an error cannot be canceled, it can be sufficiently reduced using an accurate robot dynamic model, as well as constraining the movements of the robot within the validity region of the model, e.g., applying low accelerations when a rigid body model is adopted. It must be underlined that, in a Manual Guidancesession, accelerations are kept low for safety reasons, and hence, the assumption must not be considered as restrictive in such conditions, in which Equation (2) then provides a good estimate of the torques applied by motors to counteract the external forces applied on the robot.

When the state machine in Figure 3 is in the **Monitoring** state, $I_{res}\left(t\right)$ and $\tau_{res}\left(t\right)$ are computed, and both conditions *mg_enter* and *cr_enter* are evaluated at the same time, in order to deduce the nature of the interaction between the robot and the environment.

##### Condition *mg_enter*

The detection of the condition *mg_enter* is achieved by comparing: (i) the vector of the estimated Cartesian forces applied on the end-effector $F_{mg}\left(t\right)$ with a pair of varying threshold vectors $Th1_{H}\left(t\right)$ and $Th1_{L}\left(t\right)$ and (ii) the vector $F_{mg,\;s}\left(t\right)$ of the estimated Cartesian forces slopes with a constant threshold vector $Th1_{s}$. In practice, such conditions are based on the assumptions that when a physical interaction is underway, both the Cartesian forces detected on the end-effector and their slopes increase in absolute value; such a double check approach allows making the detection procedure more reliable, so avoiding possible false detections, leading the robot to move in an arbitrary and haphazard manner.

In such a sensor-less context, the vector of Cartesian space forces $F_{res}\left(t\right)$, applied on the end-effector, could be obtained as:

(3) $$F_{res}\left(t\right)={\left(J^{T}\left(q\left(t\right)\right)\right)}^{-1}\tau_{res}\left(t\right)$$

where *J* is the Jacobian matrix and *q* is the joint position vector. In order to detect only MG-type interactions and to clean up the signal as well, a proper filtering action is required for $\tau_{res}\left(t\right)$. Observing the behavior of the residual torques while MG and CD interactions are experimentally imposed (e.g., intentionally hitting the end effector, throwing an object on it, pulling the manipulator in a smooth way or guiding it rapidly), it can be noticed that unexpected collisions produce significantly higher frequency spikes than in the manual guidance case. A low-pass filter can then be properly designed, analyzing the frequency spectrum of the force signal while MG and CD interactions are applied to the robot. Such a filter is then applied to each component of the residual current vector in (1), so obtaining the low-pass filtered residual current vector $I_{mg}\left(t\right)$, which is used to compute the estimate of the residual torques as:

(4) $$\tau_{mg}\left(t\right)=K_{t}\cdot I_{mg}\left(t\right)$$

Using Relation (3), replacing $\tau_{res}\left(t\right)$ with $\tau_{mg}\left(t\right)$ given in (4), it is possible to obtain the vector $F_{mg}\left(t\right)$, representing a good estimate of the external Cartesian forces on the end-effector as:

(5) $$F_{mg}\left(t\right)={\left(J^{T}\left(q\left(t\right)\right)\right)}^{-1}\tau_{mg}\left(t\right)$$

The slope of $F_{mg}\left(t\right)$ is then computed as:

(6) $$F_{mg,s}\left(t_{k}\right)=\frac{F_{mg}\left(t_{k}\right)-F_{mg}\left(t_{k-1}\right)}{T}$$

where *T* is the adopted sampling time and $t_{k}$ is the time instant defined as k·T. Vectors $F_{mg}\left(t\right)$ and $F_{mg,s}\left(t\right)$ are both used in the following set of conditions that are simultaneously tested to detect the *mg_enter* condition:

(7) $$\left\{\begin{array}{c} \exists \;ax\;\in \left[1,3\right]:\;F_{mg_{\left(ax\right)}}\left(t\right)>Th1_{H_{\left(ax\right)}}\left(t\right)\;\vee F_{mg_{\left(ax\right)}}\left(t\right)<Th1_{L_{\left(ax\right)}}\left(t\right)\\ \exists \;ax\;\in \left[1,3\right]:\;|F_{{mg,s}_{\left(ax\right)}}\left(t\right)|>Th1_{s_{\left(ax\right)}} \end{array}\right.$$

where ax is chosen between one and three, since only forces along the *x*, *y*, and *z* axes are taken into account, while vectors $Th1_{H}\left(t\right)$ and $Th1_{L}\left(t\right)$ are defined as:

(8) $$\left\{\begin{array}{c} Th1_{H_{\left(ax\right)}}\left(t\right)={\bar{F}}_{{res}_{\left(ax\right)}}^{N}\left(t\right)+4\sigma_{F_{\left(ax\right)}}\left(t\right)\\ Th1_{L_{\left(ax\right)}}\left(t\right)={\bar{F}}_{{res}_{\left(ax\right)}}^{N}\left(t\right)-4\sigma_{F_{\left(ax\right)}}\left(t\right) \end{array}\right.$$

where ${\bar{F}}_{res}^{N}\left(t\right)$ is the mean of the last *N* samples of $F_{res}\left(t\right)$ and $\sigma_{F}\left(t\right)$ is its standard deviation. The term $4\sigma_{F}\left(t\right)$ was inserted in the thresholds’ definition to include 99.99% of the data inside them.

Experimental choicesAn IIR low-pass filter with the cutting frequency at 2 Hz has been chosen for each joint. The structure of the adopted filter for the *j*th joint is given by:
$$I_{mg_{\left(j\right)}}\left(t_{k}\right)=a_{1}\cdot I_{mg_{\left(j\right)}}\left(t_{k-1}\right)+a_{2}\cdot I_{mg_{\left(j\right)}}\left(t_{k-2}\right)+b_{1}\cdot I_{res_{\left(j\right)}}\left(t_{k}\right)$$
where the following values have been adopted for the three parameters: $a_{1}=-1.95083889$, $a_{2}=0.95145506$, $b_{1}=0.00061616$. Thresholds $Th1_{H}\left(t\right)$ and $Th1_{L}\left(t\right)$ in (8) have been computed with *N* = 500, whereas $Th1_{s}$ in (7) has been heuristically set to 40 N/s, in order to remove false detections while leaving an acceptable level of sensitivity.

##### Condition *cr_enter*

The detection of the condition *cr_enter* is obtained by comparing the vector of filtered residual currents $I_{cd}\left(t\right)$ with a varying threshold vector $Th_{cd}\left(t\right)$, as detailed hereafter. Thresholds for collision detection are chosen as time-varying in order to avoid false detections or missed collisions.

As highlighted before, CD and MG interactions produce a quite different behavior of the force signal, which can be analyzed using its frequency spectrum. In the case of unintended collisions between the robot and the environment, high frequency spikes of force are produced, which can be detected and separated from MG signals, using a proper high-pass filter. The filter is then applied to each component of the residual current vector in (1), so obtaining the high-pass filtered residual current vector $I_{cd}\left(t\right)$.

Condition *cr_enter* is then defined as:

(9) $$\exists \;j\;\in \left[1,n\right]:I_{cd_{\left(j\right)}}\left(t\right)>Th_{cd_{\left(j\right)}}\left(t\right)$$

in which *n* is the number of joints of the robot, $I_{cd_{\left(j\right)}}\left(t\right)$ is the high-pass filtered residual current for the *j*th joint, whereas the corresponding variable threshold $Th_{cd_{\left(j\right)}}\left(t\right)$ is given by:

(10) $$Th_{cd_{\left(j\right)}}\left(t\right)=k_{cd_{c_{\left(j\right)}}}+k_{cd_{v_{\left(j\right)}}}\frac{|{\dot{q}}_{j}\left(t\right)|}{{\dot{q}}_{j,max}}+k_{cd_{a_{\left(j\right)}}}\frac{|{\ddot{q}}_{j}\left(t\right)|}{{\ddot{q}}_{j,max}}$$

where ${\dot{q}}_{j}$ and ${\ddot{q}}_{j}$ denote the velocity and the acceleration of the *j*th joint, respectively; the positive coefficient $k_{cd_{c_{\left(j\right)}}}$ is chosen heuristically for each joint, to cover the high-pass filtered currents in no-motion conditions ($\dot{q}=0$, $\ddot{q}=0$), while $k_{cd_{v_{\left(j\right)}}}$ and $k_{cd_{a_{\left(j\right)}}}$ are chosen imposing, to the *j*th joint, the maximum velocity (${\dot{q}}_{j,max}$) and the maximum acceleration (${\ddot{q}}_{j,max}$), so as to set $Th_{cd_{\left(j\right)}}\left(t\right)$ as an upper bound of the motor currents with some margin. In this way, during the standard operation of the robot, the motor currents cannot overpass the thresholds.

Experimental choicesA digital Chebyshev filter with a cutting frequency of 10 Hz has been adopted for each joint. The structure of the adopted filter for the *j*th joint is given by:
(11)$$I_{cd_{\left(j\right)}}\left(t_{k}\right)=c_{1}I_{res_{\left(j\right)}}\left(t_{k}\right)+c_{2}I_{res_{\left(j\right)}}\left(t_{k-1}\right)+c_{3}I_{res_{\left(j\right)}}\left(t_{k-2}\right)+c_{4}I_{res_{\left(j\right)}}\left(t_{k-3}\right)$$
where the following values have been adopted for the four parameters: $c_{1}=-0.239207$, $c_{2}=-0.6262528$, $c_{3}=0.6262528$, $c_{4}=0.239207$. The thresholds $Th_{cd_{\left(j\right)}}\left(t\right)$ have been obtained using the values reported in Table 1.

#### 2.2.2. Manual Guidance State

The goal of this state is the imposition of the Cartesian movement of the end-effector defined by the human operator through the forces he/she applies on the robot. The MG session finishes when condition *mg_exit* is satisfied. The conversion of the Cartesian forces $F_{mg}$ into the corresponding Cartesian positions is achieved through a proportional estimation using the theory of elasticity (only translations are taken into account) as:

(12) $$\Delta p_{mg}=K_{mg}^{-1}\cdot F_{mg}$$

where $K_{mg}$ is a diagonal matrix containing the stiffness parameters for the three main directions. Such a compliance matrix is properly set up to impose a specific behavior as a reaction; its values depend on the maximum safe speed of the robot, as well.

Experimental choicesThe compliant matrix $K_{mg}$ has been heuristically set up, in order to provide an adequate feedback to the human operator during MG sessions. After multiple tests, the value 300 N/mm has been adopted for the stiffness along every axis.

##### Condition *mg_exit*

Condition *mg_exit* is verified through a checking process in which: (i) the estimated vector of the Cartesian forces $F_{mg}\left(t\right)$ is compared with a pair of time-varying threshold vectors $Th2_{H}\left(t\right)$ and $Th2_{L}\left(t\right)$ and (ii) the mean of the estimated vector of Cartesian force slopes $F_{mg,s}\left(t\right)$ is compared with a constant threshold vector $C_{flat}$. In practice, condition *mg_exit* is satisfied when force signals are within the bounds and flattened.

The flatness of the signal is monitored considering a window of $N_{mg}$ samples, in which the average of $F_{mg,s}\left(t\right)$ should be lower than a pre-determined constant vector $C_{flat}$ in order to fulfill the flatness condition. The absolute value is also used here to include the cases of a negative slope. The values of $C_{flat}$ are critical to be determined; a high value would cancel the flatness condition, while low values would keep the system in the **Manual Guidance** state.

Condition *mg_exit* is satisfied when:

(13) $$\left\{\begin{array}{cc} Th2_{L_{\left(ax\right)}}\left(t\right)<F_{mg_{\left(ax\right)}}\left(t\right)<Th2_{H_{\left(ax\right)}}\left(t\right)\; & \forall \;ax\;\in [1,3]\\ \left|\frac{1}{N_{mg}}\sum_{k=1}^{N_{mg}}\left(F_{mg,s_{\left(ax\right)}}\left(t-k-1\right)\right)\right|<C_{flat_{\left(ax\right)}}\; & \forall \;ax\;\in [1,3] \end{array}\right.$$

where $Th2_{H_{\left(ax\right)}}\left(t\right)$ and $Th2_{L_{\left(ax\right)}}\left(t\right)$ depend on the magnitude of the force applied to the TCP, and they are computed as:

(14) $$\left\{\begin{array}{cc} Th2_{H_{\left(ax\right)}}\left(t\right)=Th1_{H_{\left(ax\right)}}\left(t\right)+c_{th2}\cdot \left|\Delta F_{mH_{\left(ax\right)}}\right|\; & for\;\Delta F_{mH_{\left(ax\right)}}>0\\ Th2_{H_{\left(ax\right)}}\left(t\right)=Th1_{H_{\left(ax\right)}}\left(t\right)\; & for\;\Delta F_{mH_{\left(ax\right)}}\leq 0 \end{array}\right.$$

(15) $$\left\{\begin{array}{cc} Th2_{L_{\left(ax\right)}}\left(t\right)=Th1_{L_{\left(ax\right)}}\left(t\right)+c_{th2}\cdot \left|\Delta F_{mL_{\left(ax\right)}}\right|\; & for\;\Delta F_{mL_{\left(ax\right)}}<0\\ Th2_{L_{\left(ax\right)}}\left(t\right)=Th1_{L_{\left(ax\right)}}\left(t\right)\; & for\;\Delta F_{mL_{\left(ax\right)}}\geq 0 \end{array}\right.$$

in which $c_{th2}$ is a pre-determined coefficient between zero and one, properly chosen to adjust the exit from the **Manual Guidance** state. $\Delta F_{mH_{\left(ax\right)}}\left(t\right)$ and $\Delta F_{mL_{\left(ax\right)}}\left(t\right)$ represent the difference between the maximum force increase after the collision and the thresholds $Th1_{H_{\left(ax\right)}}\left(t\right)$ and $Th1_{L_{\left(ax\right)}}\left(t\right)$, respectively, and are defined as:

(16) $$\left\{\begin{array}{cc} \Delta F_{mH_{\left(ax\right)}}\left(t\right)=max\left(F_{mg_{\left(ax\right)}}\left(t\right)\right)-Th1_{H_{\left(ax\right)}}\left(t\right) & \forall t\geq t_{c} \end{array}\right.$$

(17) $$\left\{\begin{array}{cc} \Delta F_{mL_{\left(ax\right)}}\left(t\right)=max\left(F_{mg_{\left(ax\right)}}\left(t\right)\right)-Th1_{L_{\left(ax\right)}}\left(t\right) & \forall t\geq t_{c} \end{array}\right.$$

where $t_{c}$ is the instant at which the interaction is detected.

In practice, when the MG session is not started yet, both upper and lower levels of Th2 are equal to the upper and lower levels of Th1, respectively, since $F_{mg}\left(t\right)<Th1_{H}\left(t\right)$ and $F_{mg}\left(t\right)>Th1_{L}\left(t\right)$. When the MG session starts, only one between $F_{mg}\left(t\right)>Th1_{H}\left(t\right)$ and $F_{mg}\left(t\right)<Th1_{L}\left(t\right)$ is satisfied depending on the direction of the change; as a consequence, only one between $Th2_{H}\left(t\right)$ and $Th2_{L}\left(t\right)$ is updated in (14) and (15), while the other one is kept at the same level. In this way, moving the arm in a certain direction will not automatically produce the same sensitivity in the opposite direction.

Experimental choicesThe best experimental results were obtained using the following parameters: $N_{mg}=50$ samples, $c_{th2}=0.7$, and $C_{flat}$ =120 N/s for each axis.

#### 2.2.3. Collision Reaction State

In this state, the system imposes a proper reaction strategy in order to move the TCP back, following the same direction of the collision force with a proportional magnitude. The collision peak can be isolated by applying a proper low-pass filter to the external force vector $F_{res}\left(t\right)$ given in (3). The so-obtained low-pass filtered force vector $F_{cr}\left(t\right)$ is then used to monitor the behavior of the force peak and to compute a proportional displacement in the Cartesian space using the theory of elasticity, previously exploited for the MG algorithm. The displacements to be imposed along the *x*, *y*, and *z* axes, collected in vector $\Delta p_{cr}\left(t\right)$, are computed as:

(18) $$\Delta p_{cr}\left(t\right)=K_{cr}^{-1}\cdot F_{cr}\left(t\right)$$

Experimental tests were carried out on different robots to analyze the force signal during collisions. They showed that in all types of collisions, the peak after an impact is attained in the subsequent 40 ms, so a good solution can be to extract the information about the direction and magnitude of the impact from the filtered force vector during this time interval.

The algorithm starts reacting directly after entering the **Collision Reaction** state. It attenuates the effect of the impact by applying a velocity profile composed of three phases:

- Phase 1: The robot position is changed according to (18). Such management is applied in the first 40 ms, acquiring the following information: (i) the time instant $t_{1}$ in which the collision is detected, (ii) the force applied in $t_{1}$, i.e., $F_{mg}\left(t_{1}\right)$, (iii) the time instant $t_{2}$ in which the force peak is reached, and (iv) the force applied in $t_{2}$, i.e., $F_{mg}\left(t_{2}\right)$. Using such information, the time interval between the collision detection and the force peak $\Delta t=t_{2}-t_{1}$ and the difference between the collision force and the force peak $\Delta F_{cr}=F_{mg}\left(t_{2}\right)-F_{mg}\left(t_{1}\right)$ are computed.
- Phase 2: A constant-speed is applied for a predefined time interval (160 ms was chosen for our implementation), by imposing at each time instant the displacement obtained using Relation (18), replacing $F_{cr}\left(t\right)$ with $F_{mg}\left(t_{2}\right)$.
- Phase 3: The stop strategy is applied. Each motor is stopped applying a deceleration profile of $N_{dec}$ samples, computed as:
  (19) $$N_{dec}=a_{s}P_{cr}+b_{s}$$
  where $a_{s}$ and $b_{s}$ are two parameters defining the type of linear relation between the deceleration time and the variable $P_{cr}$, whereas $P_{cr}$ can be defined in different ways; in particular, three alternative strategies have been tested:
  - Strategy 1: $P_{cr}=(∥K_{cr}^{-1}\Delta F_{cr}∥/\Delta t)$ (i.e., the stop interval $N_{dec}$ is a linear function of the slope of the impact force)
  - Strategy 2: $P_{cr}=∥K_{cr}^{-1}\Delta F_{cr}∥$ (i.e., the stop interval $N_{dec}$ is a linear function of the impact force)
  - Strategy 3: $P_{cr}=0$ (i.e., the stop interval $N_{dec}$ is constant and equal to $b_{s}$)

Experimental choicesAn IIR low-pass filter at 25 Hz has been adopted to filter external forces. The structure of the adopted filter for the ax Cartesian axis is given by:
(20)$$F_{cr_{\left(ax\right)}}\left(t_{k}\right)=d_{1}F_{res_{\left(ax\right)}}\left(t_{k}\right)+e_{1}F_{cr_{\left(ax\right)}}\left(t_{k-1}\right)+e_{2}F_{cr_{\left(ax\right)}}\left(t_{k-2}\right)$$
where the following values have been adopted for the three parameters: $d_{1}=0.07288762$, $e_{1}=1.46396303$, e2=−0.532685065.
For the computation of the robot displacements using (18), feasible results have been obtained using the value 120 N/mm (or greater) for all the components of the stiffness matrix $K_{cr}$. The three reaction strategies have been implemented using the values reported in Table 2.

#### 2.2.4. Waiting State

The system imposes a 1-s wait before returning to the **Monitoring** state. In this state, no movement is imposed on the robot to stabilize motor currents and to calculate decision parameters based on a no-motion situation.

### 2.3. Experimental Results

The proposed methodology has been implemented in the real industrial controller C5G of the COMAU robots, and several MG sessions and collision reaction tests have been carried out. The monitor functionality of the C5G controller has been used to perform data acquisition during MG and CD sessions. The following results are relative to a MG session carried out using a COMAU Racer 7-1.4; the data are directly provided by the C5G controller within a proper log file.

**Figure 4:**
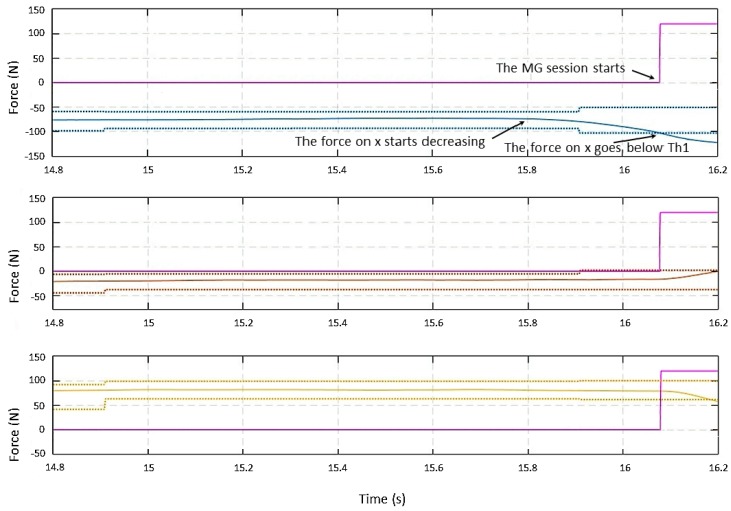
First plot from the top: filtered forces (solid blue) and Th1 on the *x*-axis (dotted blue); middle plot: filtered forces (solid orange) and Th1 on the *y*-axis (dotted orange); lowest plot: filtered forces (solid yellow) and Th1 on the *z*-axis (dotted yellow). All three plots include the transition flag (magenta).

**Figure 5:**
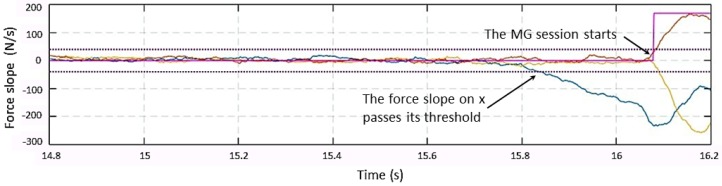
Forces’ slope on the *x*-axis (blue), forces’ slope on the *y*-axis (orange), forces’ slope on the *z*-axis (yellow), transition flag (magenta), and slope threshold (dotted violet).

As illustrated in the previous section, the collaboration session starts when at least one of the forces is greater than its threshold and when one of the force slopes achieves a predefined level. Figure 4 and Figure 5 show a manual guidance session, predominately in the *x* direction. The force $F_{mg_{\left(1\right)}}\left(t\right)$ (i.e., the external force along the *x* direction) starts decreasing at time *t* = 15.8 s, and the slope along x overpasses its threshold of 40 N/s; however, the detection happens when the $F_{mg_{\left(1\right)}}\left(t\right)$ overpasses $Th1_{L_{\left(1\right)}}$ at around *t* = 16.1 s. The plot in magenta is relative to a flag showing the transition between states; it is zero if the system is in the **Monitoring** state and different from zero if it is in the **Manual Guidance** state.

**Figure 6:**
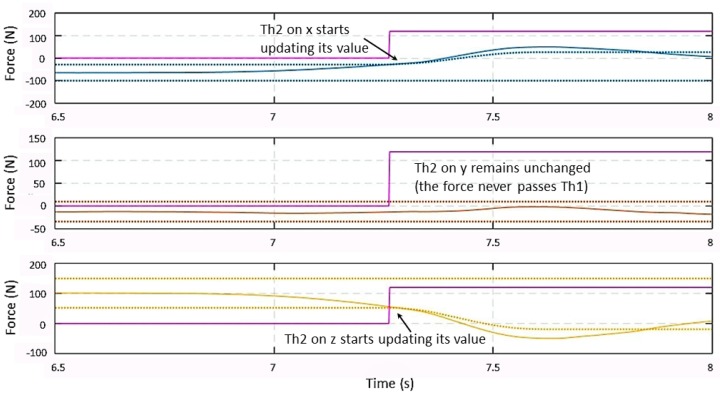
First plot from the top: filtered forces (solid blue) and Th2 on the *x*-axis (dotted blue); middle plot: filtered forces (solid orange) and Th2 on the *y*-axis (dotted orange); lowest plot: filtered forces (solid yellow) and Th2 on the *z*-axis (dotted yellow). All three plots include the transition flag (magenta).

The behavior of Th2(t), given in (14) and (15), is shown in Figure 6 after entering the **Manual Guidance** state. A force is detected on the xz plane. When the force signals along axes x and z overpass their Th1(t) thresholds, Th2(t) starts updating its value following closely the change in the force signal. Once the maximum of the force signal is reached, Th2(t) conserves its value at the same level until the end of the MG session. On the *y*-axis, the force signal does not overpass Th1(t), so Th2(t) remains equal to Th1(t).

**Figure 7:**
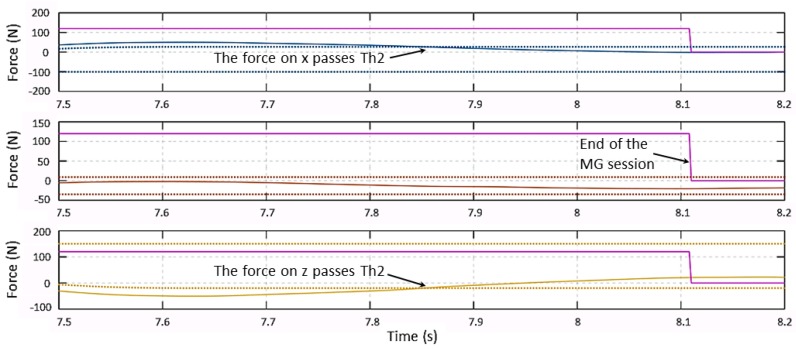
First plot from the top: filtered forces (solid blue) and Th2 on the *x*-axis (dotted blue); middle plot: filtered forces (solid orange) and Th2 on the *y*-axis (dotted orange); lowest plot: filtered forces (solid yellow) and Th2 on the *z*-axis (dotted yellow). All three plots include the transition Flag (magenta).

**Figure 8:**
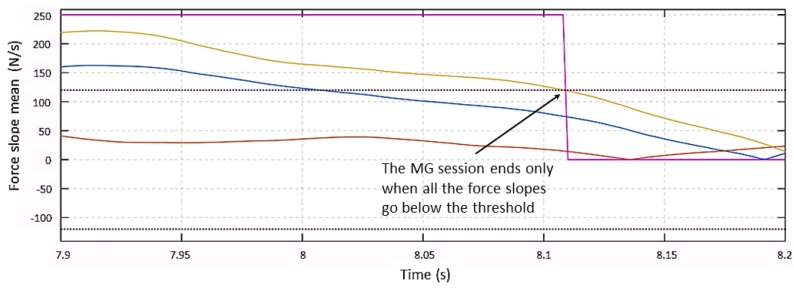
Forces’ slope on the *x*-axis (blue), forces’ slope on the *y*-axis (orange), forces’ slope on the *z*-axis (yellow), transition flag (magenta), and slope threshold (dotted violet).

After force signals go below their Th2(t) thresholds and become flat, the system returns to the **Monitoring** state. Figure 7 shows the force behavior at the end of the collaboration session. Force signals on the *x*-axis and on the *z*-axis go between their Th2 limits at 7.85 s. After such a time instant, all three signals are smaller than their thresholds, but the system does not exit the MG session until the three force slopes go below the slopes threshold at t = 8.1 s, as shown in Figure 8.

The video in [33] shows the behavior of the Racer 7-1.4 during CD and MG sessions. In the first part of the video, a post collision reaction is shown, while Strategy 2 with stiffness K = 120 N/mm is adopted.

Different collisions are applied on the end-effector and on random points of Links 4 and 5, obtaining acceptable reactions of the robot. It can be also noticed that the reaction was very good in terms of direction and safely moving away from the collision position.

The second part of the video shows an MG session. In particular (from 00:44–00:55), the ability of the low-pass filter to discard all high frequency components resulting from a fast impact and keeping all low frequency signals resulting from a normal MG interaction is highlighted.

In the third part, a second MG session is shown. In this case, at the end of the session (at 02:05), a force on Link 2 is applied, showing a slightly greater difficulty in moving the robot since a smaller leverage is applied.

In the final part of the video, a series of MG sessions is tested with a delay of 1 s between each couple of them. Forces are applied not only to the end-effector, but also to different parts of the robot. Although applying forces to the end-effector is more accurate, collisions applied on other parts of the robot gave very good results in terms of movement direction, exiting the MG session, and stopping the robot after the dissipation of the applied force.

## 3. Smart Manufacturing

The original combination of multiple sensors and actuators together with human contribution is another example of innovation and the application of the 4.0 methodologies to today’s industry. Current trends are aimed at leading the quality and repeatability levels of manual stations closer to automated cell standards.

With SMARTMAN 4.0, the authors try to demonstrate how digital systems can be used to support a worker during the execution of his/her activities, in order to optimize, monitor, and control the quality of the work in a complete way.

The objectives of this project are therefore both the streamlining of the personnel training phases and the maintenance of a constant and high quality of the produced objects.

Streamlining staff training has a strong impact in the case of small productions, concentrated in time, or that change very quickly. In these cases, reducing the time devoted to pre-production stages (e.g., for staff training) can be a significant advantage, with a view toward minimizing the costs for small production series.

With the proposed solution, the quality is guaranteed not with a retrospective control on the produced objects, but through a constant and step-by-step supervision of the entire production process. This objective is achieved by a strict control of sequences and localization in space and time of the activities of the operator, for example in manual assembly stations, logistic picking stations, or more generally whenever there is a manual station where a worker has to perform a process that needs to be verified.

### 3.1. State-of-the-Art

**Figure 9:**
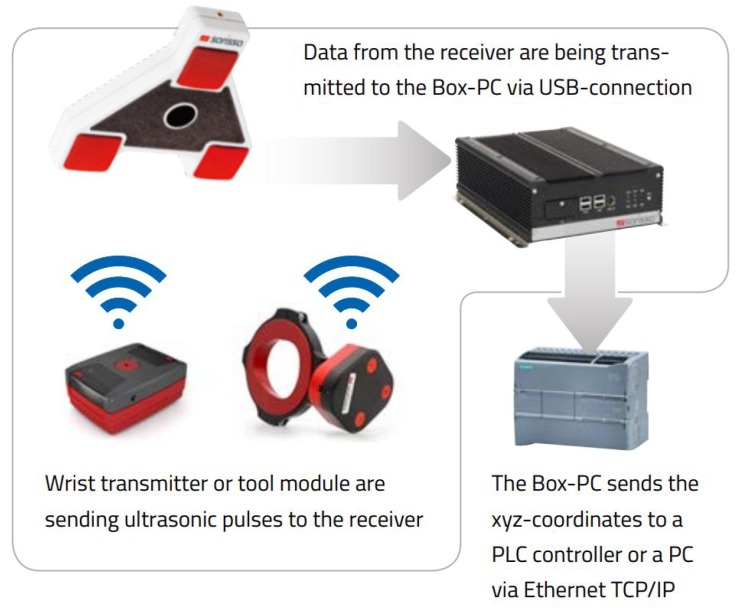
Sarissa toolkit [34].

**Figure 10:**
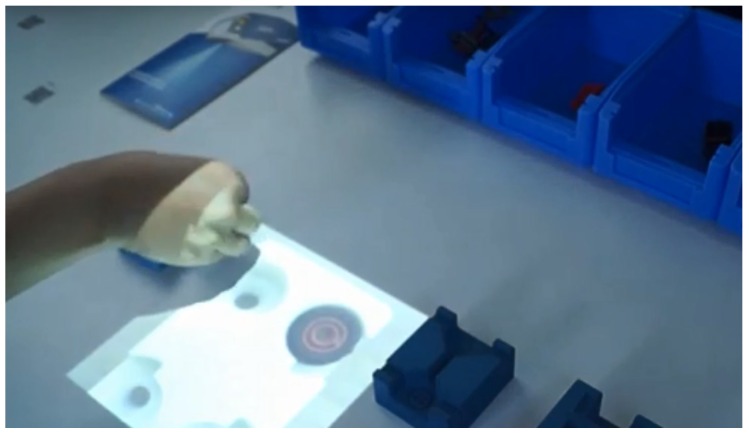
Ulixes working application.

**Figure 11:**
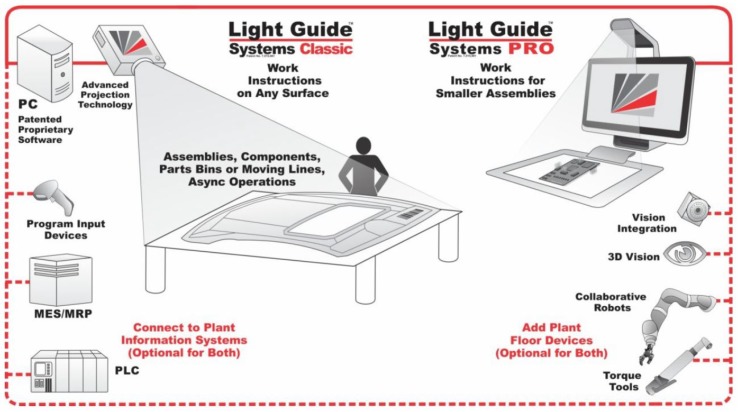
Light Guide network schema [36].

Several solutions are available on the market, possibly given by a unique, complete station or by sub-systems integrated to obtain a station. Both cases include a pointing system or a human tracking system. Some examples are briefly illustrated hereafter:

Sarissa Assistance Systems produced by Sarissa GmbH [34] is made up of ultrasonic emitters and receivers that allow locating the position of the tool in a three-dimensional environment. The receiver collects the data and sends them to a Box-PC via a USB connection. The Box-PC elaborates the information from the receiver and sends the xyz-coordinates to a PLC controller or a PC controller via Ethernet TCP/IP connection. Figure 9, taken from the Sarissa website, shows the operating scheme of such a sub-system.
Der Assistent produced by ULIXES Robotersysteme GmbH [35] mainly consists of a projection system that highlights the process steps through graphics, photos, and videos, projecting directly into the processing area and into a 3D vision system that controls the correct sequence of operations. In Figure 10, taken from a promotional video, the working area is represented during the guided operations.
Light Guide Systems produced by OPS Solutions [36] is composed of color-coded, animated light beams and visual prompts in the form of text, symbols, graphics, blueprints, or video projected on any workstation or off-line training area. This solution eliminates the operator reliance on printed work instructions, computer screens or memory for assembly guidance. Figure 11 reports the ecosystem of the solution by OPS.

The main points of comparison among the different solutions found on the market are summarized in Table 3.

### 3.2. The SMARTMAN 4.0 Solution

The proposed solution tries to be complete according to different requirements, e.g., it aims at being programmable in line, without the use of additional tools, and integrable into the line systems; moreover, it must recognize the location of the working pallet and correct any positioning errors.

**Figure 12:**
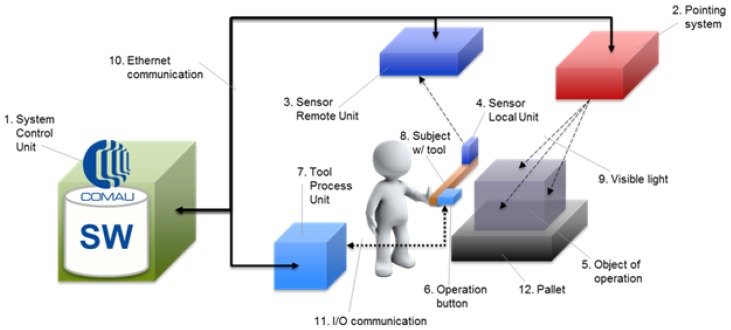
The SMARTMAN 4.0 environment.

Figure 12 shows the overall schema of the proposed SMARTMAN 4.0 environment that, starting from commercial elements without any specific customization, provides an original and innovative architecture through a smart and synergistic combination of such elements, correctly integrated with each other and properly coordinated by the developed software. This way, the proposed architecture allows overcoming the limits of the single objects and achieving a greater applicability and functionality.

The employed elements can be collected in different clusters, starting from the most general one that must be always present, up to the specific ones for a particular task, and finally to the general-purpose components that do not directly affect the quality of the final results, but simply guarantee the correct working of the architecture.

The fundamental cluster includes:

- 1. System control unit
- 2. Pointing system
- 3. Sensors remote unit

A further cluster can be put in evidence as being composed of elements devoted to the specific application as:

- 5. Object of operation
- 7. Tool process unit
- 12. Pallet

This cluster will not be analyzed in detail, being subject to the specifications of the application case and therefore not being a fundamental part of the digital station.

The items not included in these two clusters are necessary and equally important for the whole, correct working of the system, but they are given by consumer parts, like Communication Items 10 and 11.

The core objects of the system are included in the first cluster. These are the objects in which it is preferable to invest, in order to pick the best choice available on the market.

**Figure 13:**
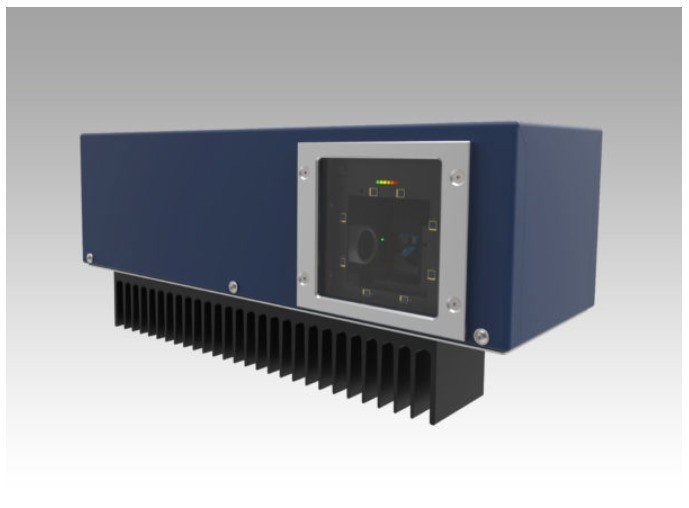
ZP1 laser.

**Figure 14:**
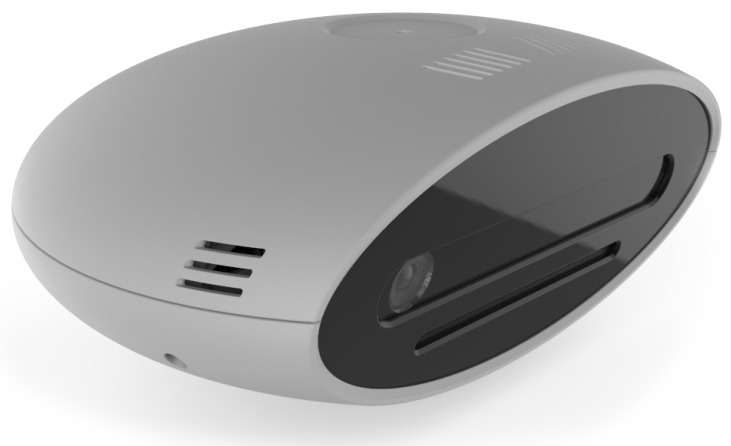
A SmartRobots camera.

System control unit: The system control unit is basically the PC, on which the necessary algorithms are implemented and which is responsible for supporting the graphical interface to the operator.
  The criteria for choosing these elements are essentially three: (i) the ability to support the hardware interfaces necessary to communicate with the laser and the camera, (ii) the possibility of easily integrating a graphic input/output device, and finally (iii) sufficient computational power to support the algorithms developed for the integration of the various sub-systems.
  The chosen PC is a panel one by B&R [37]; this solution integrates in a single device a resistive touch monitor of 15.6 inches, the necessary computing power provided by an Intel Celeron 3965S 2.2-GHz processor, and the necessary hardware compatibility to manage the Ethernet communication with the laser and the camera.
Pointing system: The pointing system is mainly dedicated to indicate to the operator where it is necessary to perform an operation and to return a visual feedback of the execution status. There are many different options on the market to project, visualize, or indicate to operators where they must act. Our research is focused on the possibility to provide additional options, mainly referring to two important aspects, from our point of view. The first one concerns the possibility of obtaining correct projections on non-coplanar surfaces, without distortions. The second one refers to the possibility of automatically re-adjusting the reference frame used for the projections, in the case of processes or supports where position and orientation may vary.
  The first characteristic is fundamental to work on complex mechanical parts, where worked surfaces are on different planes or in cases in which the surface, even if planar, is inclined to facilitate the operator’s reachability.
  The second feature arises from the need to use the solution in lines served by non-rail systems or by processes in which the positioning of the pieces is somehow arbitrary (i.e., in case of manual positioning by the human operator) and, hence, the need to find a system that not only projects images on three-dimensional supports, but which can also be rotated and translated in order to project in a consistent way, according to the positioning of the piece to be processed.
  All these features have been found in the laser pointing system produced by Z-LASER [38]. The selected laser is the ZP1, shown in Figure 13, which is the smallest laser projector in the family. It is suitable for 2D and 3D applications, safe for the eyes (laser class 2 M), and it covers working fields from 1 × 1 m up to 3.5 × 3.5 m.
  Moreover, this laser is equipped with the auto-tuning function: if reflectors are inserted on the zero plane at a known distance and geometry, the system is able to calculate the origin of the work plane, using the laser beam reflections. This information is used autonomously by the system to set the internal reference frame for the projections and is made available externally as the current origin referred to the center of the laser.
Sensors’ remote unit: In standard commercial solutions, the vision sensor aims at identifying the position of the operator’s working tool. However, to generalize the solution, we decided to trace the position of the hands instead of the one of the tool. This means that the system is able to work in a greater number of possible applications, also working where the operator has to carry out a simple gripping operation directly with the hands and is also generic for any tool without needing a phase of training for the recognition of the equipment or the insertion of specific markers for tracing the tool.
  The final requirement is therefore reduced to being able to identify and trace the hands of the operator and return their positions.
  Our choice has been to use the SmartRobots three-dimensional camera (Figure 14) [39].
  This camera processes both three-dimensional and color information and returns the position of the operator’s hands, distinguishing the right from the left. To help the system correctly identify the right operator, it was decided to let the operator wear color-coded gloves. Thanks to this simple adjustment, the camera is guaranteed to “capture” the correct worker, even in the case of several people appearing within the camera visual space.

#### Applications

The use of this architecture can be logically divided into three phases: installation, teaching, and working phase.

**Figure 15:**
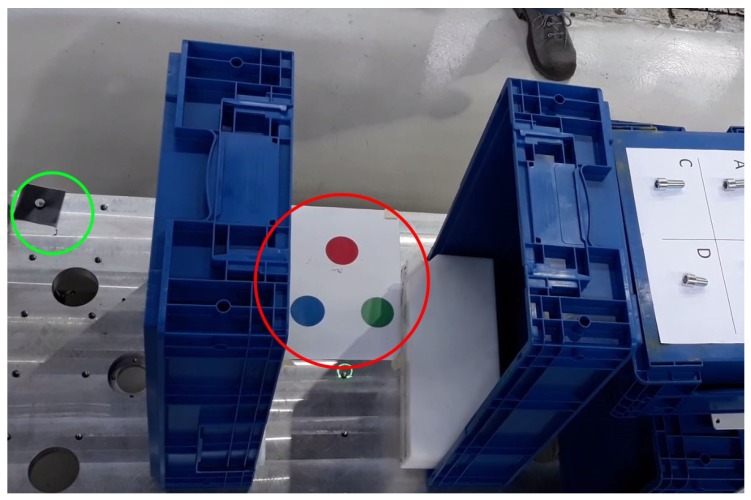
In red, the SmartRobot calibration tag and, in green, a reflector for the Z-LASER.

In the installation phase, not wanting to place heavy constraints on the geometry of the customer station, it is required that both the laser and the camera share the same operating space and that they are placed at a sufficient distance, but without imposing any relative geometric constraints. A calibration phase of the two devices is then required, to set a common reference frame. This operation has to be performed only once, at the cell installation time. The camera calibration is carried out by using a colored marker, while reflectors are adopted for the laser (Figure 15).

The teaching phase is the one during which an expert operator teaches the system the set of operations and the relative sequence. This phase is carried out every time it is necessary to add a new sequence in the station or it is necessary to add or remove steps by modifying a previously-acquired sequence.

**Figure 16:**
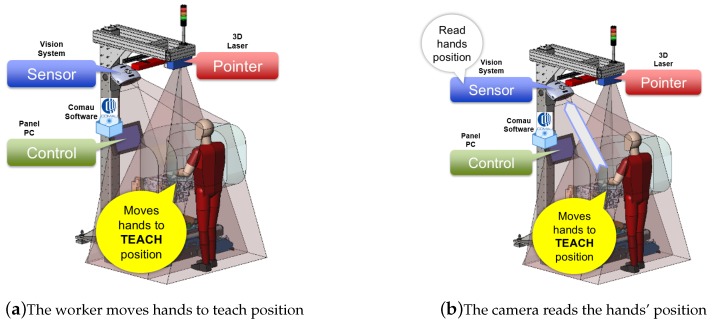

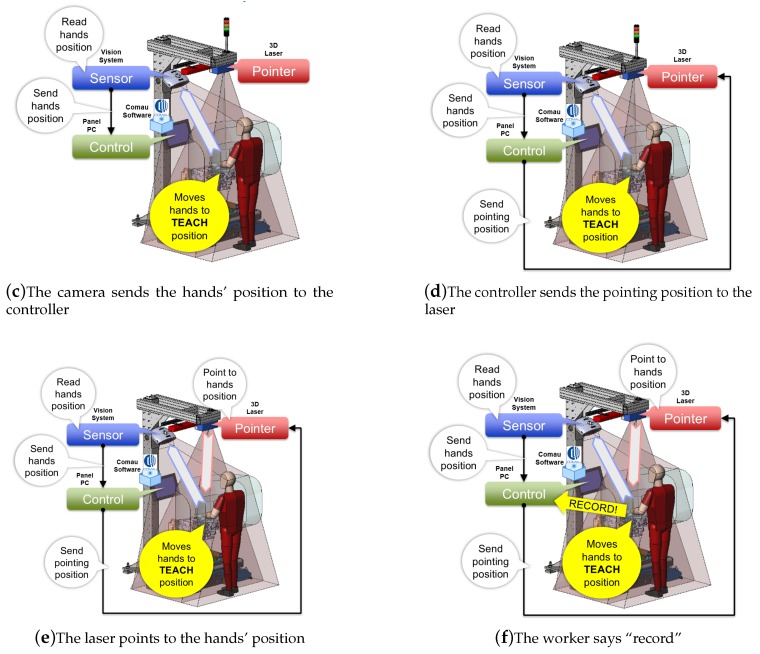
Workflow for the recording phase.

The workflow for recording a point of a sequence is reported in Figure 16 and summarized hereafter:

- The operator puts the hands in the correct configuration to execute the first step of the task (Figure 16a).
- The camera recognizes the position of the hands (Figure 16b).
- The camera sends over TCP/IP the current position of the hands to the control unit (Figure 16c).
- The control unit, once having obtained the hand position, uses the calibration information and sends a target position to the laser (Figure 16d).
- The laser points over the hand of the worker to confirm the read position (Figure 16e).
- The worker says “record” (Figure 16f).
- The system confirms the acceptance of the command, by drawing a special graphical symbol on the operator’s hands.

In the last step of this sequence, we have added a phase in which the operator commands the station to record the position by means of a voice command. This option arose from the observation that for certain operations, it is probable that both hands of the operator are engaged, and it is equally probable that, during the teaching phase, the operator may want to settle the most correct pose. It was therefore decided to add to the control unit the basic software necessary to perform speech recognition.

The working phase is the one in which the system guides the operator along the correct sequence of operations, checks the correct positioning, and only in the case of congruence, enables the equipment to perform the task, thus generating an interlock between the correct sequence and manual activity, ensuring a constant quality of production.

**Figure 17:**
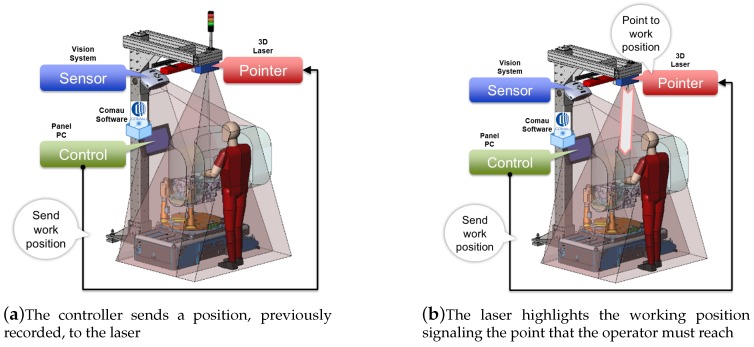

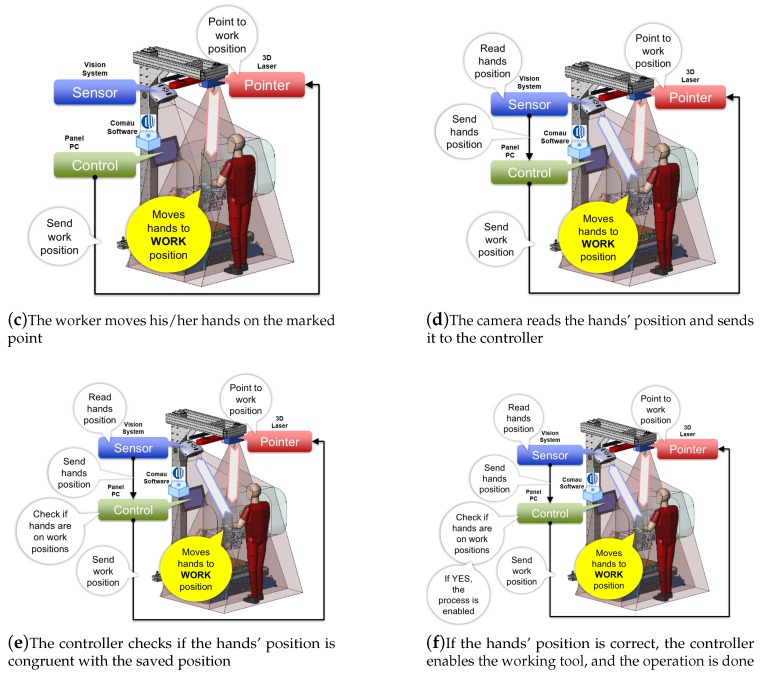
Workflow for the working phase.

The workflow for executing a step of a recorded sequence (see Figure 17) is:

- The controller sends a position to the laser (Figure 17a).
- The laser points to the working position (Figure 17b).
- The worker moves his/her hands to the working position (Figure 17c).
- The camera reads the hands’ position and sends it to the controller (Figure 17d).
- The controller checks if the hands’ position is correct (Figure 17e).
- If the hands’ position is correct, the laser draws a special symbol on the operator’s hands, a vocal message is given, and the working tool is enabled (Figure 17f).

In this sequence, there are no direct interactions between the operator and the control unit if everything is done correctly; in case the operator takes too long to perform the operation, the system signals a time-out. In the event of a time-out, the operator can set the repetition of the step or the abortion of the whole sequence, again, via voice interface. Particular attention has been paid to the HMI (Human-Machine Interface) issues. The use of graphic symbols directly drawn on the worker’s hands, as a feedback during teaching and working phases, along with the speech recognition and synthesis, enhance the ergonomics of the station. Thanks to the adopted solutions, the operator is not forced to shift his/her gaze, each time focusing elsewhere on HMI supports, or to make wearying continuous movements of the neck.

The described system can therefore be positioned, with respect to the state-of-the-art, as indicated in Table 4, which puts in evidence the completeness of the SMARTMAN 4.0 solution.

The management of the different operating modes takes place through a simple software selection, which does not require physical interventions in the field. This means that it is possible to modify, add, or remove points and sequences quickly and dynamically, allowing the management of even minimal production flows, without affecting the correctness of operations and an exhaustive quality control.

This type of innovation is probably the one that comes closest to the Industry 4.0 paradigm, based not on deep vertical technical knowledge, but on an overall view of the process, needs, and characteristics. It is basically the creation of a sensor system, or more briefly over-sensor, born from the intelligent fusion of information and specific capabilities of individual existing objects, which is going to improve, in an alternative way, a complex and structured reality, without the need for a long complex and expensive development. A smooth transition from the current industrial methods toward a fully-integrated 4.0 reality is simplified by the design of a modular sub-system to be easily added to existing items.

The modularity of the sub-system can be exploited in the future also for new major additions in a production cell, increasingly oriented toward cooperation between humans and collaborative machines.

## 4. AMRs as Meta-Sensors

The main goal of the on-going HuManS project (including COMAU, Politecnico di Torino, and several industrial and academic partners) is the creation of a new manufacturing paradigm, in which the human operator is at the center of the production system, by means of innovative technical solutions that allow the execution of highly-complex operations, through a safe and efficient interaction among operators, robots of different natures, and working stations. The authors of this paper directly involved in the project deal in particular with the development of an advanced robotic architecture for a flexible production line, in which an AMR fleet acts as a meta-sensors’ net, integrating the AGVs’ knowledge of the environment, so as to avoid the use of a fixed sensor net that would imply infrastructural interventions, limiting the flexibility of the line itself. The proposed architecture is illustrated in this section, after a general overview about the state-of-the-art solutions and AMR definition.

### 4.1. State-of-the-Art and AMR Definition

The mobile component of an industrial setup was originally restricted to manually-driven forklifts [40]: autonomy and efficiency degrees have been then gradually improved by the introduction of solutions based on AGVs that however lack on-line decision capabilities, in the presence of unexpected obstacles. AGVs, classified as logistics professional service robots in manufacturing environments [41], represent the traditional setup in modern warehouses and factories.

AGVs follow fixed paths (roadmaps), and their motion is thus dependent on the factory/warehouse infrastructure: roadmaps are predefined and not flexible, which implies that a small change in the setup may cause long inactivity periods. Less flexibility then represents a risk for productivity performances and possible economic losses.

As with traditional industrial robots, i.e., industrial manipulators, the AGV’s immediate surroundings are, in principle, off-limits to human operators. The current and future trend aims at “setting free” all industrial robots, allowing human-robot cooperation by de-caging the manipulators and by slowly getting rid of the virtual cage enclosing the AGVs. The evolution of conventional AGVs goes toward AMRs, generically indicating infrastructure-free, highly-autonomous mobile platforms, which position themselves as a solution to industrial flexibility issues.

**Figure 18:**
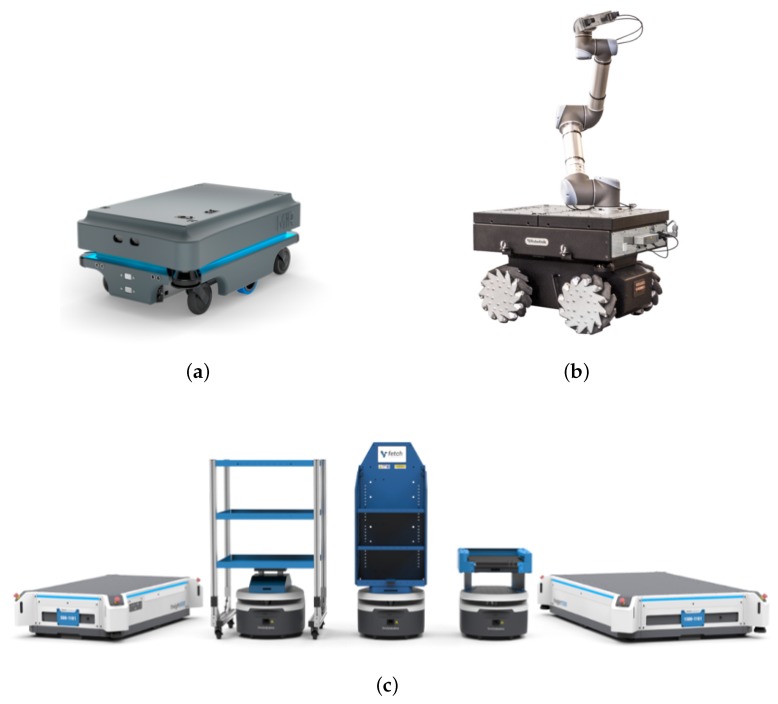
Examples of AMRs. (**a**) The MiR200 is a safe, cost-effective mobile robot. It can be equipped with customized top modules, including lifts, bins, and cobots [45]. (**b**) The RB-KAIROS is a completely integrated Collaborative Mobile Manipulator (CMM), designed for the development of industrial tasks [46]. (**c**) Fetch robot AMRs’ collection [47].

Giving a unique and unambiguous definition of AMR is currently very difficult due to the current development of the concept, which depends on the field of application and operational requirements. Nevertheless, in order to highlight the features that characterize the new concept of AMR (in an industrial context), the following criteria are pointed out:

Degree of autonomy One of the main characteristics of the AMR is the capability of understanding its surroundings and being able to navigate dynamically. Indeed, the presence of on-board, more advanced systems allows for sophisticated decision-making capabilities [42]. An AMR is, at its most autonomous version, capable of avoiding static and dynamic obstacles, including human operators. The degree of autonomy may vary, based on the requirements.
Degrees of freedom The definition of AMR not only includes a “simple” moving platform (as standard AGVs), but may include other systems on top of it extending the autonomous mobile robot DOF. Indeed, mobile manipulators, i.e., mobile robots coupled with a robot arm on it, are used for many industrial operations that can involve cooperation with human operators [43]. Thus, the presence of additional automated structures extends an AMR’s capabilities and application possibilities (Figure 18).
Application Depending on the robot kinematics and sensors’ set, the applications can vary. For example, a mobile manipulator can support factory workers with repetitive and precision industrial processes in a flexible way, transferring the production line where needed (this is not possible with fixed-base cobot arms, i.e., collaborative robot arms). Mobile robots are employed in logistics tasks (e.g., the transport of different loads between areas) that can be dull, repetitive, and/or hazardous for human workers. Autonomous mobile robots are also used for moving products and goods between workers and stations, allowing one to minimize idle time. Another utilization is maintenance tasks in restricted access areas and inventory processes with predefined schedules, allowing for optimized inventory process times in huge warehouses [44]. Furthermore, some AMRs can implement person-following skills, docking to machinery, and voice control.
Ease of integration An automated guided vehicle follows fixed routes, usually along wires or magnets embedded in the ground. This implies that a small change in the working space configuration is constrained by the AGVs’ road-paths: adding a new AGV to an existing fleet is a difficult process, and extending the working space for the AGVs is not immediate. On the other hand, AMRs are not dependent on the infrastructure since they dynamically determine the best route according to pre-learned maps. In this way, AMRs’ motion does not depend on the environment setup and allows for fast integration in the factory/warehouse workflow, without the need for expert staff or any re-layout.
Scalability As a consequence of the ease of integration of AMRs in a factory setup, the number of smart mobile robots can be increased without being hindered by structural changes. A system of AMRs is thus highly and easily scalable.
Safety standards As already mentioned, autonomous mobile robots are accessing spaces usually reserved for human operators and manned vehicles. Indeed, with respect to AGVs and, obviously, the traditional fixed automation elements, the AMRs are free to roam around: no fences and predefined routes imply major safety concerns. In this regard, the drawing up of new tailored safety standard guidelines is currently underway: safety guidelines for AMR may be a fusion between safety compliance standards for AGVs and human-robot interaction specified in collaborative robots standards (ISO/TS 15066:2016-Robots and robotic devices – Collaborative robots).
  The Robotic Industries Association (RIA) together with the American National Standards Institute (ANSI) are currently developing safety standards for industrial mobile robots, aiming at publication in 2019 [48]. As regards mobile platforms, the closest existing standard is ANSI/ITSDF B56.5-2012, Safety Standard for Driverless, Automatic Guided Industrial Vehicles and Automated Functions of Manned Industrial Vehicles. This standard “defines the safety requirements relating to the elements of design, operation, and maintenance of powered, not mechanically restrained, unmanned automatic guided industrial vehicles and the system of which the vehicles are a part” [49]. As stated, this standard is defined for classical AGVs and does not take into account the higher degree of autonomy of current AMR systems: no real-time path re-planning and optimized routing is considered.
Artificial intelligence Even though the motion environment of an AMR is overall structured (factory/warehouse map), it is dynamic due to moving platforms and factory workers. Consequently, the AMR needs a set of sensors whose information, intelligently fused, is the input to sophisticated algorithms providing artificial intelligence to the platform. Object detection, surroundings’ perception, and many other capabilities are critical for the AMR’s autonomy.
Cost The use of AMR in preexisting workspaces does not imply an infrastructure re-configuration, which translates into a less costly integration. Furthermore, newer technologies result in faster and cost-effective performances. Therefore, AMR solutions are overall less expensive with respect to traditional AGV systems.

Having defined the characteristics of an AMR, some projects can be considered as important reference points for the transition from traditional AGVs to AMR systems.

In [50], the mobile robot, the environment, and planning were modeled as automata, exploiting modular supervisory control theory. In this way, the robot is able to navigate in the presence of unpredictable obstacles: two supervisors guide the mobile robot enforcing the path it has to follow while ensuring collision avoidance, task management, and static versus dynamic obstacles. The research presented in [51] aims at applying cyber-physical systems to the design of AGV systems and fosters efficiency, taking care of urgent tasks, allowing overtaking. In this system, AGVs become smart agents thanks to the physical layer where perception, decision-making, and communication are implemented with sensors, a computing module, and a Wi-Fi module, respectively.

In [52], the Plug and Navigate (PAN) Robots project is presented, which aims at providing an advanced logistic system involving smart AGVs. These AGVs are able to compute their motion autonomously along a set of virtual paths, called a roadmap, pre-computed using a semi-automated map creation process [53]. The mobile platforms are capable of overtaking obstacles found on (or near) the predefined path, exploiting local deviations [54]: the detouring operation is assisted by a centralized data fusion system that gathers together on-board sensing and the data output from an infrastructure-based environment perception system.

The autonomous mobile robot is thus an evolution of the automated guided vehicle, adapted to the imperative need of the industrial evolution to break the now obsolete and inefficient fixed production line paradigm. Moreover, the AMR’s main intelligence lies in the sensor data fusion, fostered by the use of the latest sensing technologies. Therefore, the AMR sensing system should represent an added value not only for the AMR platform itself, but for the whole smart factory ecosystem, made up of inter-connected, on-line, real-time devices.

In the work-in-progress project presented in this section, the idea is to exploit the mobile platforms as a distributed sensor infrastructure, and as a support net to existing traditional AGV systems. The aim is to design a system of AMRs taking on the role of meta-sensors, going beyond the distinction between smart entities and sensing systems, at a higher level of abstraction.

### 4.2. The Proposed Architecture

The system under development is thought for ideally any flexible production line, made up of classical AGVs, workstations, and cobots, in spaces accessible to human operators. The system can be described by inspecting the macro-elements composing it:

1. meta-sensor AMR fleet
2. sensors’ synergy center
3. AGV coordination center interface

At a high-level, we can identify the target system as an Industry 4.0 sensor system, supporting non-autonomous agents. Note that, in this case, the word “sensor” comprehends both sensors and meta-sensors.

The meta-sensor entities integrate the AGVs knowledge about their surroundings, allowing reacting smartly to foreseeable approaching dynamic obstacles, e.g., factory workers. The aim is to provide real-time fully-conscious reactions to the changing environment, which would be unachievable with a fixed sensor net.

Information gathered from the AMRs is combined together within a data fusion center, called the Sensors’ Synergy Center (SSC), which generates an overall updated picture of the plant traffic status. Furthermore, an ad-hoc interface allows translating significant data into commands, to modify motions planned by the AGV Coordination Center (AGV CC) accordingly. Note that the AGV CC represents the generic pre-existent classical AGV manager. Communication between the two centralized systems (SSC and AGV CC) is bi-directional: indeed, the AGV CC can forward task requests to AMRs.

#### 4.2.1. AMR as Meta-Sensor

As mentioned above, the meta-sensor AMR aims at increasing the traditional AGV consciousness about obstacles on its route, especially when blind intersections are involved. In this case, the AMR is a sort of AGV extension, an integral part of its sensor system: it is a meta-sensor, whose activity is related to the following main issues.

**Figure 19:**
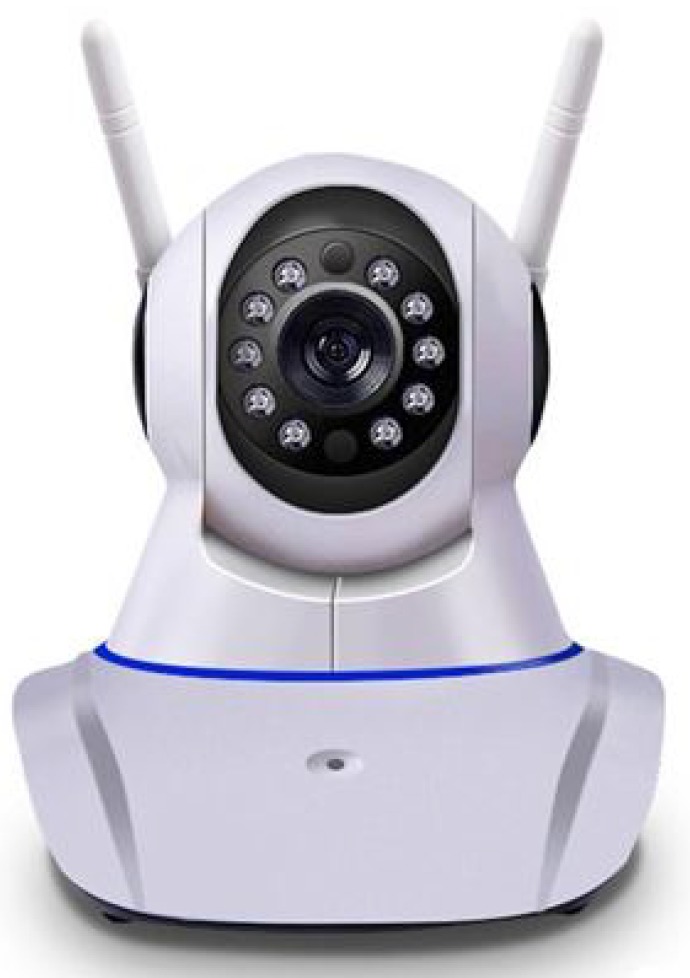
The use of low-cost IP cameras is under inspection, as off-the-shelf sensors for computer vision algorithms.

Navigation The AMR exploits the ROS (Robot Operating System) [55] navigation stack, which uses relationships between coordinate frames, sensor values, and odometry information to perform planning, obstacle avoidance, and at will, localization in a previously-created map, using the KLD-sampling (adaptive) Monte Carlo localization approach [56]. The global planner can implement the Dijkstra or the A* algorithm, while the vehicle task allocation is entrusted to the preexistent AGV CC in order to integrate the AMRs according to known and tested rules.
Vision Recent computer vision developments provide object recognition and detection, fundamental components for sorting out the appropriate reaction for mobile robots.
  A benchmarking to find the best option for the vision information is currently going on: conventional solutions are compared to new, unexplored alternatives. FireWire (IEEE1394a or IEEE1394b) cameras are well supported by ROS, which provides an image pipeline useful for camera calibration and raw image low-level processing, e.g., distortion rectification and color decoding [57]. In order to get depth information, a stereo vision camera could be a choice. However, the combination, fusion, of two or more sensors can produce a more robust piece of information, since disadvantages can be neutralized and advantages summed up. A solution under evaluation is combining a laser range finder for depth information, with a monocular camera. In the specific analyzed solution, we have a low-cost PTZ IP camera (Figure 19).
  Currently, PTZ IP cameras can be easily set up since they usually come with a plug-and-play application, which lets the user connect the camera to the preferred wireless access point Wi-Fi signal. An IP camera is a standalone unit, accessible via its IP address. The majority of IP cameras are compatible with the ONVIF protocol, a set of web services-based specifications, using open standards, e.g., XML, to determine how communication occurs between electronic devices over an IP network [58]. The IP camera RTSP (Real-Time Streaming Protocol) output stream has been captured using the GStreamer tool [59], a pipeline-based media processing framework, and processed so as to obtain a jitter-free, de-payloaded, RGB stream, re-directed as a Linux virtual video device. This video has been read as an ordinary video device, using the ROS tool gscam [60], which, leveraging GStreamer, can attach itself to a specially-formatted pipeline. Thanks to gscam, the stream can be broadcast as a standard ROS image message. In this way, the image is processed through the ROS image pipeline and can be accessed by other ROS distributed nodes (e.g., the SSC processes). Moreover, the low level parameters of ONVIF-compatible devices can be accessed and modified using Python libraries, making it possible to manage via ROS the motion of this kind of camera. Other options, in combination with laser scanners, will be evaluated, such as omni-directional cameras for a wider field of view and stereo cameras, to allow depth information redundancy.
Safety As concerns safety measures, the idea is to complement safety-compliant sensors (e.g., safety-rated laser scanning systems) with non-conventional and/or low-cost sensors that may not be intrinsically safe, but provide an overall secure piece of information, as a result of sensor data fusion, performed within the SSC.
Network communication A further key enabler of advanced manufacturing is wireless communication. Wireless networks comply with many Industry 4.0 requirements, e.g., operational flexibility and easier setup, unlike wired alternatives. Cost-effectiveness is another advantage supporting the low-cost and high-performance aims. Since we consider a factory/warehouse scenario, we expect to work with indoor robots, allowing us to consider a Wi-Fi connection for smart elements’ intercommunication.
  For this aim, ROS provides a well-documented network setup, where all distributed nodes are networked via a local Wi-Fi router: robot data are shared among nodes within the same network, through ROS communication paradigms (e.g., ROS topics) [61]. However, ROS’s heavy dependency on TCP/IP standards leads to issues, e.g., reliability problems, bandwidth limitations, jitter, and delays [62]. With regard to these challenging network problems, ROS2 [63] promises to ensure native real-time and multi-robot performance-enhancing features, through the use of Data Distribution Service (DDS) as the networking middleware. However, given its state of initial development, ROS2 does not represent a robust choice, at the moment.
  Thus, ROS can be a good network choice for the prototyping of the foreseen system, but obviously with the knowledge that (i) there are limitations and shortcomings in the network communication management, which need to be compensated for (through the implementation of ad-hoc nodes or external support frameworks making up for deficiencies) during the development of the real implementation and (ii) emerging technologies, e.g., 5G networks and 5G architectures, aimed at meeting latency, resilience, coverage, and bandwidth requirements, possibly overcoming present issues [64].
  In its “Guide to Industrial Wireless Systems Deployments” [65], NIST (National Institute of Standards and Technology) provides manufacturers and users with best practice guidelines, depending on operating requirements and environments. These guidelines have been established following a joint industrial wireless workshop with IEEE, exploring the latest and future wireless technologies [66]. The guide points out key features on which to select the proper wireless network, e.g., network throughput, addressing method, reliability, safety, and size of the system (number of device), which will be for sure taken into account for the project development.

#### 4.2.2. Sensors’ Synergy Center

Sensors’ data from each meta-sensor AMR are gathered in a centralized system where information is processed to obtain appropriate control commands that, sent to the AGV CC through the dedicated interface, regulate the AGVs’ motion according to the identified objects. The sensor fusion process comprehends both intra-AMR sensors fusion and inter-AMR data fusion, returning an overall overview of the plant state.

Currently, images are gathered and input in the advanced state-of-the-art, real-time, object detection algorithm YOLO (You Only Look Once) v3 [67], in order to identify objects and accordingly perform decisions. YOLO applies a single neural network to the full image, dividing it into regions and predicting bounding boxes and probabilities for each region. Detection can be performed using a pre-trained model (on the COCO detection dataset [68]), and computations can be performed on a GPU, since the used neural network framework, the open source Darknet framework [69], is written in C and CUDA.

The SSC has the aim of representing a centralized computation node, allowing unloading heavy computations from distributed agents. The AGV CC receives the plant traffic overview while informing the SSC about the AGV poses, allowing for object matching. Moreover, when needed, the AGV CC can send task allocation requests, for tasks that may not be completed by AGVs due to unavailability or too-long of a waiting time. Indeed, a meta-sensor AMR, while still providing crucial sensing information, can assist a human operator in collaborative operations or to perform urgent tasks, if requested. Note that tasks should be assigned to the AMR if the requirements are within its payload and/or precision capabilities.

A possible implementation for information transmission between computational centers would involve the network communication intrinsically provided by ROS.

**Figure 20:**
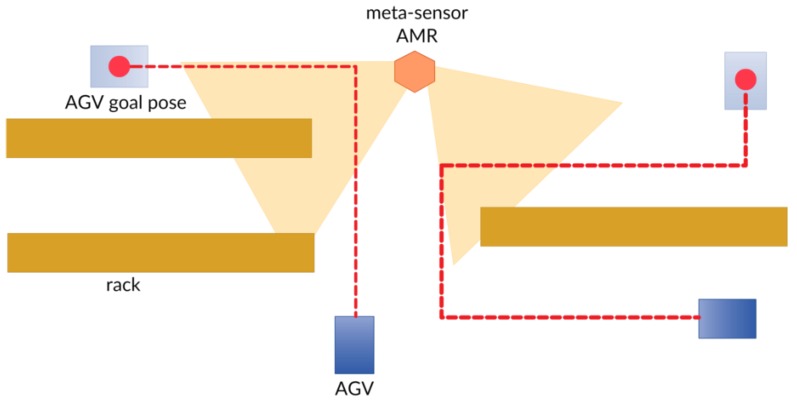
The AMR is sent in the neighborhood of blind intersections, which are foreseen to be traveled by AGVs.

**Figure 21:**
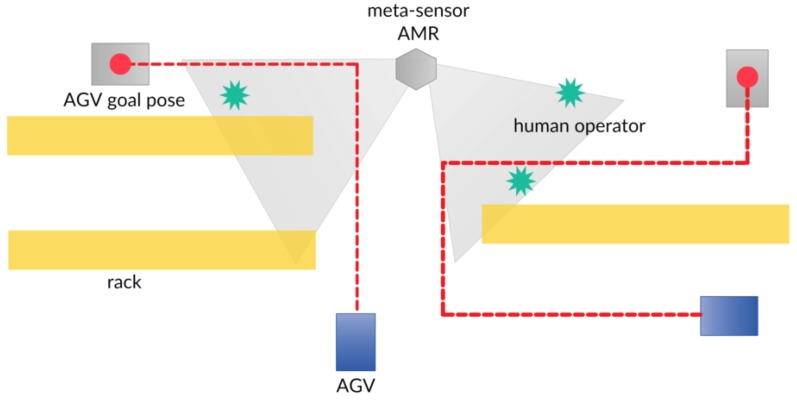
The AMR can detect human operators and obstacles (e.g., racks) and inform the SSC, which will warn the AGVs (through the AGV Coordination Center (CC)), just in time.

The SSC addresses the AMR towards spots where AGVs are supposed to travel (Figure 20), according to the route allocation provided by the AGV CC, so as to ensure a safe passage, by advertising the presence of an obstacle/human operator (Figure 21).

#### 4.2.3. AGV Coordination Center Interface

In order to enable communication between the SSC and the AGV CC, an interface has to be implemented. Keeping in mind the intention of using ROS as a software development framework, the idea is to exploit the message-based communication paradigm to easily map information from the AGV CC to the SSC. Examples can be the AGVs poses translated into “tf” ROS messages and a task allocation request implemented as an ROS action. On the other hand, the plant traffic overview output by the SSC must be translated into information useful for the AGVs’ route selection. The AGV CC interface should be made up of adaptable methods, translating data formats from one system to the other. The SSC side can be kept untouched, while the AGV CC interface side implementation depends on the pre-existent coordination system in the plant where the meta-sensor system has to be deployed.

### 4.3. Remarks and Future Works

**Figure 22:**
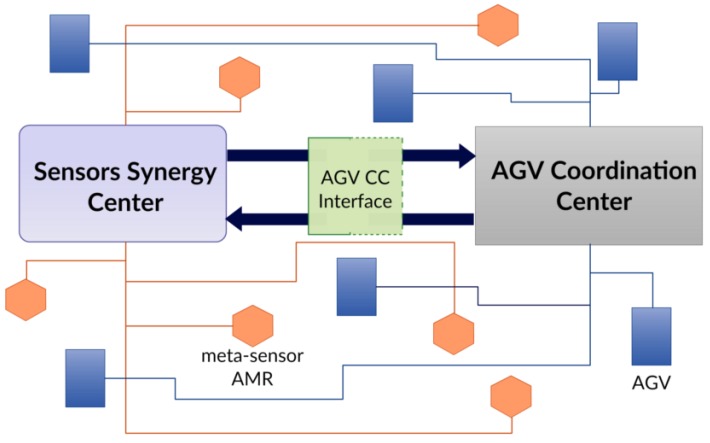
Interactions going on in the foreseen system. Communication between the SSC and the AGV CC is bi-directional: the former generates the overall plant traffic map, by gathering data from the AMR fleet, while the latter updates the AGVs poses in the SSC traffic map and deals with the AGVs coordination.

In Figure 22 are represented the dynamics at play within the meta-sensor and AGV systems.

The aim of the system under development is to enrich the factory/warehouse ecosystem with a net of autonomous mobile agents, the meta-sensors, serving the role of blind intersection sentries. Each meta-sensor is equipped with safety-rated sensors, whose data are combined with possibly non-conventional low-cost vision sensors’ data. The system aims at exploiting data fusion and open source tools to achieve an optimized system behavior, representing a solution within anyone’s reach. In future works, the system’s main element, i.e., the meta-sensor AMR, could be treated as an abstract set of features, leveraging ideally any kind of hardware and software frameworks.

## 5. Final Discussion and Conclusions

In this paper, three different combinations of existing sensors were presented, obtaining results superior to the mere union of the parts, in order to make today’s industry, despite its limits, in line with the most modern and advanced principles of Industry 4.0.

In the first part, it was shown how using the standard on-board current sensors of a manipulator, it is possible to obtain a different and intuitive human-machine interface, in order to facilitate the interaction between automatic and human systems. This kind of result provides an immediate, significant upgrade in all the programming phases of any manipulator, independently of the specific application involved.

In the second part, the use of common commercial sensors combined in a different way supports the stations where a manual process takes place. In this case, the union of the sensors has more simultaneous functions, i.e., quality control, operator’s guide through the operational steps, and improvement of the ergonomics of the station itself. The potentialities of the proposed solution are quite broad, since its adoption may be advantageous not only for the performances of the industrial process, thanks to a reduction of the staff training time and to the possibility of a strict, online quality control of the parts produced in manual stations, but also for the human operator, whose activities are facilitated in a user-friendly way.

In the third part, the concept of the industrial environment is dealt with in a broader way; in this case, the use of several AMRs located in the plant and the centralized use of data obtained from multiple sensors on several AMRs leads to the complete sensorization of the plant. The goal here is to speed up the adaptation of the current industrial plants to the requirements of the smart factories’ scenario, avoiding invasive infrastructural interventions.

These three examples therefore illustrate how the principles of Industry 4.0 can be reached with punctual operations, without the need to start from a green-field for their implementation, but through a wise use of already existing sensors, the targeted addition of sensors, and the use of a different concept of automation that can be gradually applied to existing situations. The practical adoption of the three contributions has already begun, just starting from the production line of robots. The SMARTMAN 4.0 solution is in fact internally adopted by COMAU itself in the production lines of its manipulators (in particular in the assembly of the wrist). Once the manipulators are ready to be inserted in properly-designed robotic cells, the manual guidance procedure can provide a useful aid to the integrators during the entire programming phase. After the setup of a production line including various robotic cells and manual stations, the combination of the three proposed solutions can enhance its flexibility to face possible changes in the production, since (i) the re-programming phases are facilitated by the manual guidance procedure; (ii) the maintenance of high quality standards for the manual stations can be achieved thanks to the SMARTMAN 4.0 solution, avoiding too long training phases of the staff; (iii) the AMRs’ fleet provides the possibility of easily rearranging the production scenario.

The applications, for which the most significant benefits are envisaged by the synergistic adoption of the three proposed solutions are relative to production lines providing a small number of items per hour, which require manual interventions in several phases to guarantee very high standards. An example can be given by the production of cars of high quality and cost, in which benefits can be achieved not only by the manual guidance procedure for an accurate programming of the automatized stations and by the adoption of SMARTMAN 4.0 for the manual ones, but also by the employment of AMRs instead of classical forklifts, to improve safety in an environment characterized by a high presence of human operators.

## Figures and Tables

**Table 1:** Parameters values of the varying threshold function $Th_{cd}\left(t\right)$ in the experimental implementation.

	Joint					
Parameter	1	2	3	4	5	6
$k_{cd,c}$ (A)	0.45	0.4	0.25	0.05	0.05	0.05
$k_{cd,v}$ (A)	0.2	0.2	0.1	0.04	0.03	0.02
$k_{cd,a}$ (A)	0.02	0.04	0.03	0.02	0.01	0.01
${\dot{q}}_{max}$ (rad/s)	0.05	0.06	0.09	0.32	0.32	0.36
${\ddot{q}}_{max}$ (rad/s${}^{2}$)	0.003	0.004	0.0045	0.01	0.01	0.01

**Table 2:** Values of $a_{s}$ and $b_{s}$ for the three reaction strategies experimentally applied.

	Strategy 1	Strategy 2	Strategy 3
$a_{s}$	7500	281	any
$b_{s}$	−250	−62	500

**Table 3:** Comparison of current manual station digitalization solutions.

Feature	*Sarissa*	*OPS Solution*	*Ulixes*
*Lights*	False	2D Beamer	2D Beamer
*Sensors*	3D Ultrasound	False	3D Vision
*Tool Tracking*	3D Ultrasound	False	False
*Gesture/Speech Recognition*	False	False	False/True

**Table 4:** Comparison of current manual station digitalization solutions and SMARTMAN 4.0.

Feature	*Sarissa*	*OPS Solution*	*Ulixes*	*SMARTMAN 4.0*
*Lights*	False	2D Beamer	2D Beamer	3D Laser
*Sensors*	3D Ultrasound	False	3D Vision	3D Vision
*Tool Tracking*	3D Ultrasound	False	False	3D Vision
*Gesture/Speech Recognition*	False	False	False/True	True

## Abbreviations

The following abbreviations are used in this manuscript:

AGV	Automated Guided Vehicle
AMR	Autonomous Mobile Robot
CD	Collision Detection
FSM	Finite State Machine
HRC	Human-Robot Collaboration
MG	Manual Guidance
TCP	Tool Center Point
PC	Personal Computer
USB	Universal Serial Bus
PLC	Programmable Logic Controller
TCP/IP	Transmission Control Protocol/Internet Protocol
HMI	Human-Machine Interface
DOF	Degrees Of Freedom
SSC	Sensors’ Synergy Center
AGV CC	AGV Coordination Center
ROS	Robot Operating System
KDL	Kullback–Leibler Distance
PTZ	Pan-Tilt-Zoom
ONVIF	Open Network Video Interface Forum
XML	Extensible Markup Language
RTSP	Real-Time Streaming Protocol
RGB	Red-Green-Blue
DDS	Data Distribution Service
YOLO	You Only Look Once
COCO	Common Objects in Context
GPU	Graphics Processing Unit
CUDA	Compute Unified Device Architecture
